# Supplementary material for: Identification of photoperiod-induced specific miRNAs in the adrenal glands of Sunite sheep (Ovis aries)
Source: Front Vet Sci. 2022 Jul 22;9:888207. doi: 10.3389/fvets.2022.888207 (PMC9354845; doi:10.3389/fvets.2022.888207)
Supplement: Supplementary file 1 [file Data_Sheet_1.ZIP › Supplementary file/miRDeep2 file.zip!/miRDeep2 file/Know_Structure/LP42A2/oar-mir-380.pdf]

miRBase precursor : oar-mir-380
Total read count : 2867
oar-miR-380-5p read count : 32
oar-miR-380-3p read count : 2833
remaining reads : 2

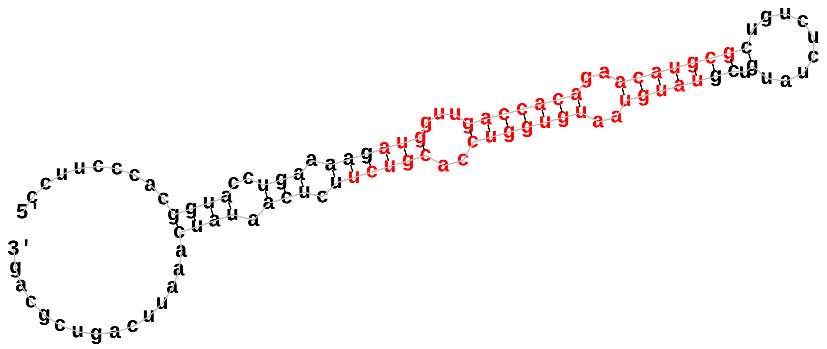

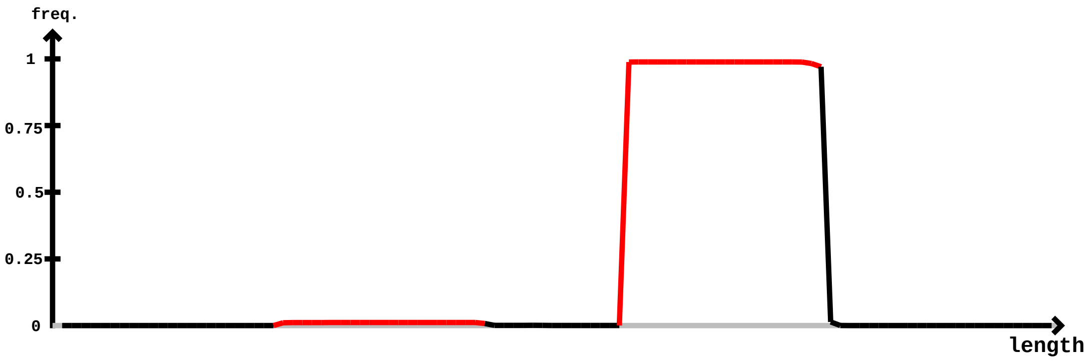


```
                                     oar-miR-380-5p
                                                                      oar-miR-380-3p
5'- ccuucccacgguaccugaaaagaugguugaccacagaacaugcgcugucucuaugucguauguaaugugguccacgucuucucaauaucaaauucagucgcag   -3'    exp
    .........((((..(((.((((((...(((((((..((((((((.........).)))))))..)))))))..)))))).))).))))..............    reads      mm          sample
    ......................augguugaccacagaacaugc............................................................    10         0           seq
    ......................augguugaccacagaacaugcg...........................................................    19         0           seq
    ......................augguugaccacagaacaugcgcuguc......................................................    1          0           seq
    .......................ugguugaccacagaacaugc............................................................    1          0           seq
    .......................ugguugaccacagaacaugcg...........................................................    1          0           seq
    ...........................ugaccacagaacaugcgcugucu.....................................................    1          0           seq
    ................................................cucuaugucguauguaaugugguccacguc.........................    1          0           seq
    ..........................................................uauguaaugugguccacg...........................    1          0           seq
    ..........................................................uauguaaugugguccacgu..........................    16         0           seq
    ..........................................................uauguaaugugguccacguc.........................    34         0           seq
    ..........................................................uauguaaugugguccacgucu........................    2742       0           seq
    ..........................................................uauguaaugugguccacgucuu.......................    38         0           seq
    ..........................................................uauguaaugugguccacgucuuc......................    1          0           seq
    ...........................................................auguaaugugguccacgucu........................    1          0           seq
```
